# Criteria for assessing high-priority drug-drug interactions for clinical decision support in electronic health records

**DOI:** 10.1186/1472-6947-13-65

**Published:** 2013-06-13

**Authors:** Shobha Phansalkar, Amrita Desai, Anish Choksi, Eileen Yoshida, John Doole, Melissa Czochanski, Alisha D Tucker, Blackford Middleton, Douglas Bell, David W Bates

**Affiliations:** 1 Partners Healthcare Systems, Inc., 93 Worcester Street, 2nd Floor, Wellesley Gateway, Wellesley, MA 02481, USA; 2 Division of General Medicine and Primary Care, Brigham and Women’s Hospital and Harvard Medical School, 1620 Tremont Street, Boston, MA 02120, USA; 3 RAND, 1776 Main Street, Santa Monica, CA 90401, USA; 4 Department of Medicine, University of California Los Angeles, 405 Hilgard Avenue, Los Angeles, CA 90095, USA; 5 University of Chicago School of Medicine, Chicago, IL 60637, USA

**Keywords:** Clinical decision support, Drug-drug interaction, Medication-related decision support system, Electronic health record, Alerts

## Abstract

**Background:**

High override rates for drug-drug interaction (DDI) alerts in electronic health records (EHRs) result in the potentially dangerous consequence of providers ignoring clinically significant alerts. Lack of uniformity of criteria for determining the severity or validity of these interactions often results in discrepancies in how these are evaluated. The purpose of this study was to identify a set of criteria for assessing DDIs that should be used for the generation of clinical decision support (CDS) alerts in EHRs.

**Methods:**

We conducted a 20-year systematic literature review of MEDLINE and EMBASE to identify characteristics of high-priority DDIs. These criteria were validated by an expert panel consisting of medication knowledge base vendors, EHR vendors, in-house knowledge base developers from academic medical centers, and both federal and private agencies involved in the regulation of medication use.

**Results:**

Forty-four articles met the inclusion criteria for assessing characteristics of high-priority DDIs. The panel considered five criteria to be most important when assessing an interaction- Severity, Probability, Clinical Implications of the interaction, Patient characteristics, and the Evidence supporting the interaction. In addition, the panel identified barriers and considerations for being able to utilize these criteria in medication knowledge bases used by EHRs.

**Conclusions:**

A multi-dimensional approach is needed to understanding the importance of an interaction for inclusion in medication knowledge bases for the purpose of CDS alerting. The criteria identified in this study can serve as a first step towards a uniform approach in assessing which interactions are critical and warrant interruption of a provider’s workflow.

## Background

Medication-related decision support (MDS) alerts generated at the point of prescribing have the potential to prevent adverse drug events (ADEs) and improve patient safety [1]. Despite their potential benefit, these alerts are often ignored [2] as a result of ‘alert fatigue’ [3]. Alert fatigue is the consequence of receiving a high volume of alerts whereby users start ignoring critical alerts along with those that may be clinically insignificant [4-7]. Studies evaluating the reasons for these high override rates have found that the interactions for which the alerts are generated often lack clinical significance and may also have inadequate literature evidence supporting their validity [4-6]. Previous studies have identified over-alerting as a barrier to EHRs achieving their full potential in improving patient safety [6,7].

The Stage 1 Meaningful Use (HIT) certification standards required the implementation of drug-drug and drug allergy checking in EHRs [8]. In Stage 2, the recommendation was for EHRs to employ interaction checking -drug-drug and drug-allergy with the ability for the provider to refine DDI rules. In Stage 3, the criteria may well call for a nationally endorsed lists of high priority DDIs to be implemented in EHRs [9]. A major limitation in being able to develop such a list of high-priority DDIs was the lack of consensus on criteria that should be used to evaluate these interactions in order to prioritize them as clinically significant. While the significance of an interaction is largely dependent on the clinical context in which it presents itself, such as the patient’s status, medication profile, etc., there are a number of criteria that need to be evaluated when an interaction is considered for inclusion in a medication knowledge base. Currently, knowledge base vendors and in-house curators of knowledge bases employ disparate criteria, with varied relevance, resulting in a lack of consensus and low overlap on what are considered the most severe DDIs [10]. We employed a mixed approach, first, assessing the state-of-the-art in terms of DDI assessment as described in the literature. Second, we validated these criteria with a panel of experts, both from the content and implementation perspective to assess both the validity and the feasibility of utilizing these criteria for the assessment of DDIs in medication knowledge bases used in EHRs.

In 2010, the Office of the National Coordinator for Health Information Technology (ONC-HIT) commissioned an effort to address the challenges of alert burden and its impact on EHR adoption [11]. The larger effort was aimed at identifying key interactions that should be implemented in knowledge bases to reduce alert fatigue [12]. A first step in identifying these interactions was to assess what criteria these interactions need to meet in order to be deemed as high severity DDIs. The specific goal of this study is to describe important criteria for choosing interactions to include for CDS in an EHR.

## Methods

We conducted a 20-year systematic literature review, from 1990 to 2010 of two bibliographic databases, MEDLINE and EMBASE, in order to identify articles that described characteristics of high-priority DDIs. The criteria identified from the review were then validated by a panel of subject matter experts (SMEs) representing various perspectives in the domain of medication decision support in EHRs - EHR vendors, academic medical centers, and regulatory agencies, such as the Food and Drug Administration and professional organizations such as the American Society of Health-System Pharmacists. No human subjects or data from human subjects was used in this study.

### Systematic literature review

**Figure 1:**
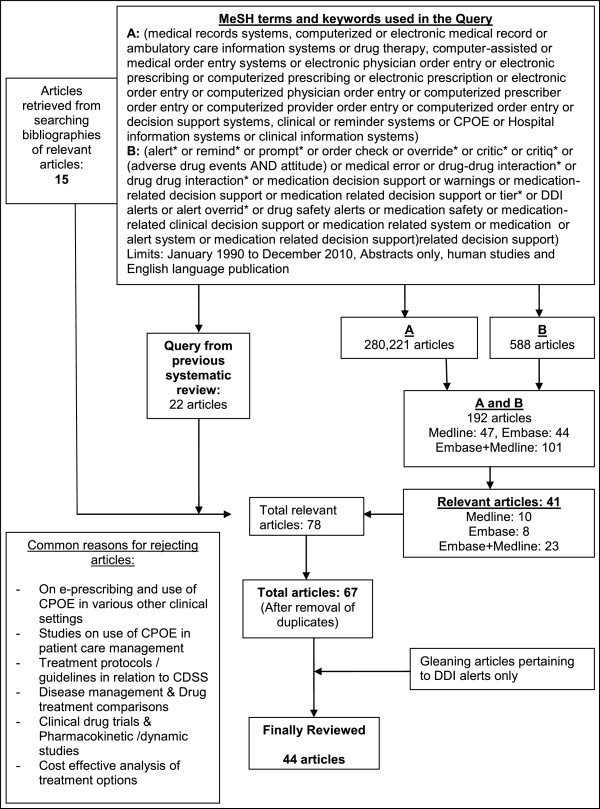
**Methodology for extracting relevant articles in the systematic review process.** Provides an illustration of the methodology used in conducting the systematic review. The review focused on extracting articles on medication-related decision support in electronic health records.

Two literature databases, MEDLINE and EMBASE were searched using keywords and MeSH terms for relevant studies on medication related decision support in clinical information systems. A detailed list of terms employed for this search is listed in Figure 1. The search was restricted to the time period of January 1990 to December 2010. We looked for the presence of specific keywords and MeSH terms in two categories in the title, abstract, and body of the articles. The categories employed were as follows:

i) Category (A) consisted of terms that described a clinical information system or electronic medical record.

ii) Category (B) consisted of terms referring to medication-related decision support interventions including mechanisms (alerts, warnings, reminders, etc.) of generating MDS.

Using the keywords identified in categories A and B we identified 280,221 and 588 articles respectively. An intersection of these two sets of terms [A AND B] resulted in 192 articles which contained concepts from both sets of keywords. We reviewed the abstracts for these articles and identified 41 articles that matched our inclusion criteria. In addition, we looked at the bibliographies of “relevant articles”, which yielded 15 articles and a previously conducted systematic review on the topic of alert fatigue [4] identified 22 articles resulting in a total of 78 relevant articles. After removing duplicates, we had a total of 67 articles which were independently reviewed. Following a comprehensive review, of these articles, reviewers identified 43 articles that matched our criteria. Common reasons for excluding articles at this stage included: articles on e-prescribing and use of CPOE in various other clinical settings, use of CPOE in patient care management, treatment protocols/guidelines in relation to CDSS, disease management & Drug treatment comparisons, clinical drug trials & pharmacokinetic/pharmacodynamic studies, and cost effective analysis of treatment options. See Additional file 1 for a list of the 43 articles reviewed. Two reviewers assessed all articles that matched these inclusion criteria to determine relevance. Following this first round of elimination, a team of five reviewers, consisting of 3 pharmacists, 1 physician and 1 human factors expert, all with expertise in clinical information systems, reviewed the relevant articles to extract criteria for high priority DDIs. Each article was reviewed by at least three reviewers, one of whom (SP) served as a moderator to resolve differences in the criteria extracted by the primary reviewers. Each reviewer independently extracted criteria from relevant studies on how the evidence on DDIs could be filtered or tailored to identify the high priority, clinically significant DDIs.

### Expert panel discussions

Experts with a diverse background in the development, maintenance and implementation of medication-related decision support in EHRs were invited to participate on a panel to validate the criteria and barriers in implementing a list of high priority DDIs. Owing to the diversity of their expertise and the institutions that they represented, the panelists provided viewpoints from the perspective of a variety of relevant stakeholders. Expert panelists represented the following categories:

• 6 participants representing 5 academic medical centers (University of Washington, University of Washington Medical Center, Arizona CERT, Columbia University, and Erasmus University Medical Center)

• 5 participants representing 5 commercial knowledge vendors (Thompson Reuters, Wolters Kluwer, Cerner Multum, First Data Bank and Lexi-Comp)

• 2 participants representing 2 organizations with in-house medical knowledge base maintenance and development (Partners Healthcare and the Veterans Administration)

• 2 participants representing federal or private agencies involved in the regulation of medication use (The Food and Drug Administration (FDA) and The American Society of Health-System Pharmacists (ASHP))

**Figure 2:**
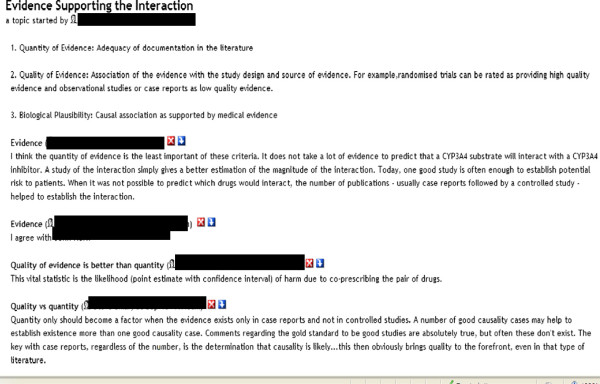
**Example of the discussion between expert panelists on the criteria of the ‘Evidence Supporting the Interaction’.** Names associated with specific comments have been hidden to maintain the anonymity of the contributors. Provides a screenshot of the virtual discussion portal used by the expert panel for assessing criteria identified from the literature review.

In addition, we conducted individual phone calls with representatives from the three leading medication knowledge base (KB) vendors [Cerner Multum, First Databank, and Wolters Kluwer]. These phone calls provided in-depth discussion of the criteria identified via the literature review but also allowed discussion of practical considerations for the use of these criteria by knowledge base vendors in assessing DDIs. Additionally, the expert panel was provided the opportunity to discuss the criteria using an online portal called eRoom (EMC, version 7). An example of the discussion between expert panelists on the criteria of the ‘Evidence supporting the interaction’ is illustrated in Figure 2. Names associated with specific comments have been hidden to maintain the anonymity of the contributors.

The conversations during the calls were recorded with the permission of the participants and transcribed verbatim for analysis. Panelists were not provided any financial incentive for participation and were chosen because of their expertise in the area of medication related decision support, especially drug-drug interactions in the context of use in medication knowledge bases for CDS in EHR systems.

## Results

The criteria identified from the systematic literature review and validated with the panel are presented in Table 1. These criteria represent what the literature indicates are important considerations for how to choose which DDIs to include in an EHR. In addition, by validating these criteria with the medication knowledge base vendors we were able to arrive on barriers for employing these criteria and provide considerations for how they should be employed in order to improve DDI alerting in EHRs.

**Table 1:** Criteria for identifying clinically important drug-drug interactions for clinical decision support in electronic health records

**Criteria**	**Description of the criteria and key sub-criteria that emerged from the literature review and expert panel discussion**
1. Severity of interaction [13-15]	■ Clinical Importance: Hansten, Horn and Hazlet in their ORCA classification identify clinical importance as a function of both the inherent danger of the drug combination and the extent to which the presence of risk factors predisposes the patient to the interaction. Also consideration of potential severity of the adverse outcome (ORCA classification, Hansten, et al. [14])
	■ Likelihood of Mortality
	■ Likelihood of Morbidity
	■ Likelihood of Intervention: The probability of the suggested intervention being able to prevent harm caused by the interaction.
2. Probability of interaction [13,14]	■ Likelihood of the Adverse Reaction
	■ Timing of Administration
	■ Consideration of the pharmacokinetic properties of the interaction: Some studies such as Siedling, et al. have evaluated pharmacokinetic characteristics of DDIs between statins and various drugs. The study revealed that more than half of the concentration-dependent ADEs related to statins were considered inappropriate if the upper dose limits were taken into account.
	■ Dose and Duration of Therapy
	■ Route of Administration
	■ Sequence of Administration
	■ Monitoring planned for the patient
	■ Therapeutic window of the object drug
	■ Combination of drugs commonly used for therapeutic reasons
3. Clinical implications [14,16-18]	■ Management burden: defined as the course of action a clinician may have to take for each potential drug interaction
	■ Monitoring planned for the interaction
	■ Awareness of the intervention: Likelihood that providers may be aware of the ability to intervene in order to prevent harm caused by an interaction.
4. Patient characteristics [13,14,18]	■ Taking into account alcohol, diet, smoking and drug use which might alter the characteristics of the drug in consideration resulting in possible DDIs.
	■ Importance of age
	■ Importance of gender
	■ Concurrent diseases
	■ Other active medications on the patient's profile
5. Evidence supporting interaction [13-15,19-21]	■ Quantity of evidence: Adequacy of documentation in the literature
	■ Quality of evidence: Association of the evidence with the study design and source of evidence. For example, randomized trials can be rated as providing high quality evidence and observational studies or case reports as low quality evidence.
	■ Biological plausibility: Causal association as supported by medical evidence

Provides a descriptive representation of the criteria used to identify clinically important drug-drug interactions. This list focused on five categories, which include Severity of interaction, Probability of interaction, Clinical implications, Patient characteristics, and Evidence supporting interaction.

The panel considered five criteria to be most important when assessing an interaction- Severity, Probability, and Clinical Implications of the interaction, Patient characteristics, and the Evidence supporting an interaction. In order to further assess each criterion, there are several sub-criteria that need to be taken into account. Twenty-four sub-criteria were identified for the 5 criteria: 4 for Severity of Interaction, 9 for Probability of Interaction, 3 for Clinical Implications, 5 for Patient characteristics, and 3 for Evidence Supporting the Interaction. We have listed these in Table 1 and identified the citations from which the sub-criteria were derived. The panel also identified barriers and considerations in order to use these criteria in medication knowledge bases used by EHRs, which are described in Table 2.

**Table 2:** Barriers and considerations for including clinically important drug-drug interactions (DDIs) in electronic health records (EHRs)

**Barriers for the use of standardized criteria for identifying DDIs for use in EHRs**	**Considerations suggested by the expert panel**
1. Large disparities between drug knowledge bases and among local experts [22,23]	The overlap between what is deemed as clinically significant by different knowledge bases is low. Besides the disparity across databases there is often disagreement among local experts (depending on clinical expertise and role) on the list of critically important DDIs.
2. Resource intensive process	The process of conducting literature reviews and vetting them with users of the EHR is a resource intensive process. Not all organizations have the ability to expend clinical resources in order to customize their knowledge bases from a commercially supplied DDI set. The knowledge management committee might need to re-evaluate the customized DDI set at the time of every update provided by the vendor.
3. Need for ongoing review	The list of clinically significant DDIs must be reviewed periodically in order to keep the knowledge base current. This involves conducting ongoing literature reviews to assess whether the evidence surrounding a DDI has changed since the time it was first assessed as critically important.
4. Lack of context of patient populations [24]	Knowledge base vendors lack the ability to contextualize DDIs based on the specific patient populations where the EMR is used. Some guidelines can be provided in order to improve specificity (e.g. for a geriatric population) however the use of these guidelines is limited and dependent on the clinical practice where the EMR is implemented.
5. Inability to alert on DDIs caused by discontinuation of drugs [25]	DDI alerts are based on drugs that are co-prescribed or administered together. Knowledge base vendors are unable to provide DDI alerts for an interaction that may be caused by the discontinuation of a drug. For example, the drug combination of clonidine and propranolol where the discontinuation of clonidine from combined therapy with propranolol may produce elevation of blood pressure.
6. Close integration with patient data in EMR	Several patient characteristics play an important role in being able to identify the set of clinically significant DDIs. While these patient characteristics, such as age, gender, or specific lab values, are known to knowledge base providers they cannot be readily implemented because these require close integration with the EHR in which the knowledge base is used. Since KB vendors do not have control over the use, expression or standardization of these data elements, their consideration in filtering the list of DDIs is limited.
7. Implementation of strategies to reduce “alert fatigue” based on physician responses	One mechanism of reducing "alert fatigue" is taking into account previous responses of the user to an alert. For example, if a physician has already seen an alert for a DDI should the same alert be shown upon renewal of the medication? Additionally, if a physician has previously determined a particular drug combination to be appropriate for a patient then should he/she be re-alerted when renewing the drug combination for the same patient? The inability to account for physician responses limits KB vendors from providing solutions that take into account provider responses in the EHR system.
8. Customizing DDI list based on clinical workflow	Consideration of the clinical workflow can also help streamline the alerts seen by clinicians. For example, certain DDI alerts can be shown only to nurses since these would occur only if the administration times of the medications were close together or where the sequence of administration of the drugs is important. In this scenario, the physician need not be alerted. It is difficult for knowledge base providers to implement such mechanisms of streamlining the sub-set of DDIs shown to specific providers.
9. Software sophistication	The sophistication of the software necessary to implement a DDI into a CDS system that also considers patient information i.e. lab results is a challenge when it comes to space and the upkeep necessary to such a system.

Provides a descriptive representation of the barriers associated with including clinically important drug-drug interactions. This list focused on nine categories, which include Disparities between knowledge bases and local experts; Resource intensive process, Need for ongoing review, Lack of context of patient populations, Inability to alert on DDIs caused by discontinuation of drugs, Close integration with patient data, Implementation of strategies to reduce alert fatigue, Customizing DDI list based on clinical workflow, and Software sophistication.

### Criteria for developing a list of clinically important drug-drug interactions

#### Severity of the interaction

Tiering alerts on the basis of severity is one of the most important measure to reduce alert overriding and was discussed in detail [26]. Experts agreed that severity of the interaction should primarily be related to the risk/benefit of using the drug pair concomitantly. Weighing the potential benefit would require careful consideration of patient and disease characteristics. Clinical information that provides context for an interaction is not readily available to knowledge base vendors thus their assessment of interactions accounts for generic situations rather than specific patient variables. In addition to the assessment of the risk/benefit based on clinical context, one panelist opined that when easy to use alternatives are available, one accepts less risk and therefore "severity" for such an interaction should be greater. More research is needed to understand this variation for assessing the severity of an interaction.

For the more practical consideration of how many levels of severity should exist, the literature review showed that there is no clear consensus on class designation and terminology. There is also a lack of a developed taxonomy for stratifying drug-drug interaction alerts, although a five-category operational classification has been suggested [13]. The Veterans Administration system classifies DDIs as “critical” or “significant,” [27] while First DataBank identifies three levels of DDIs- “contraindicated”, “severe”, and “moderate” [28]. This is much larger than a problem of semantics since the interpretation of these labels varies and no clear definitions exist on how DDIs fit into one category versus another. While some medication knowledge base vendors do have clear definitions, these distinctions are seldom obvious or available to the end-user who is presented with the interactions.

#### Probability of the interaction

According to the panelists the likelihood of causing harm is just as important as severity. However, it is harder to determine the probability without knowing the patient context. In order to assess the likelihood of an adverse drug event (ADE), panelists suggested including the concentration response curve for the ADE of interest. This is especially important when the ADE is unrelated to the desired pharmacologic outcome, e.g., seizures caused by the use of an analgesic such as meperidine [29]. Consideration of the timing of administration is also important in determining whether an interaction would actually take place. By adjusting the administration times in the medication administration record to account for the half lives of the drugs, a time-dependent drug-drug interaction can be prevented. For example, the interaction between quinolones and multivalent cations, resulting in the decreased absorption of the former, can be avoided by a rule requiring separation in administering the drugs [30,31]. The formulation of the drug should be considered as another sub-criterion to rule out those which occur due to specific inactive ingredients present in certain formulations, e.g. alcohol, which can interfere with the release mechanism of sustained-release morphine causing too much of the drug to be released rapidly resulting a potentially fatal dose. In addition, the pharmacokinetic properties of the drugs should also be evaluated to rule out presentation of inappropriate alerts. Seidling, et al. evaluated 73 high risk statin-drug combinations for their pharmacokinetic properties and concluded that more than half of the DDI alerts that presented in a clinical decision support system were inappropriate since the DDI-specific upper dose limits were not taken into account [32].

#### Clinical implications of the interaction

Panelists suggested consideration of the management burden of the interaction, the monitoring planned and the awareness of the provider regarding the interaction. Management burden is defined as the course of action a clinician may have to take for each potential drug interaction. Hansten and Horn describe three types of actions- a. considering alternative drugs that may be less likely to interact and thus avoiding the interaction b. circumventing the interaction by minimizing the consequence of the interaction and c. monitoring the patient to detect the consequence of the interaction and minimizing the adverse outcome. For example the interaction between the aripiprazole, a drug used for the treatment of the symptoms of schizophrenia, and the antiarrhythmic agent, amiodarone can be managed in three ways. First, since the interaction occurs due to amiodarone’s ability to inhibit the enzyme CYP3A4 responsible for the metabolism of aripiprazole, another anti-arrhythmic agent which does not display this inhibitory effect, should be considered. For example, calcium, channel blockers other than diltiazem and verapamil are unlikely to inhibit CYP3A4. Second, if the provider decides to co-prescribe the two drugs, the dose of aripiprazole should be reduced to one-half of the usual dose and if the inhibitor is subsequently withdrawn then the dose should be adjusted back to the usual dose. In addition, the patient should be carefully monitored for aripiprazole-related adverse reactions, such as shaking, vomiting or confusion and alter the dose if such effects are observed. Third, monitoring of the plasma concentration of aripiprazole is also recommended when used in combination with amiodarone [33]. Previous research conducted by Hines and Warholak on DDI alerts seen in pharmacy information systems, showed that over 35% of the systems evaluated in the study, did not provide strategies for managing an interaction [34]. This is an important piece of information that allows the clinician to make the right decision based on patient context. In the absence of information on clinical consequence of co-prescribing two interacting drugs providers would not be able to gauge the severity of the interaction as it relates to a specific patient.

#### Patient characteristics

Specificity of alerting can be improved by developing rules that take into account patient specific data. This can be achieved by close integration between the medication knowledge base and the EHR. By extracting patient specific variables from the EHR, the alert will be more meaningful and allow the physician to only see alerts relevant to the patient at hand. For example, accounting for the glomerular filteration rate or the creatinine clearance to check for compromised kidney function or the potassium level would be relevant before firing an alert for an interaction between an antihypertensive medication such as a beta blocker (labetalol and metoprolol) and an angiotensin converting enzyme inhibitor (lisinopril, enalopril and captopril) [35]. According to Krall *et al*. [16]. the CDS should also allow rapid and easy updates to a patient’s information to ensure continuous relevant alerts as per their condition [36]. In future, when EHRs are more mature in handling genetic characteristics, these could also be accounted for in further improving the specificity of the alerts fired for a patient. Medication knowledge base vendors pointed out that estimating likelihood of an interaction in the general population is already hard, thus to write executable rules which take into account patient characteristics listed would be even more challenging and expensive, though it may be feasible in the future. Further, existing gaps in terminology and standardization of knowledge representation along with absent context specific algorithms make connections between the KB and the EHR difficult to achieve currently. In light of these difficulties it was suggested that either the corresponding alert text should mention some of these pieces of information or the information should be made available on demand (e.g., by providing a link) [37]. The supporting information should be visually distinct from the main message of the alert and brief so that it can be easily interpreted or ignored when not needed.

#### Evidence supporting an interaction

The quantity and quality of the evidence associated with an interaction which relates to the adequacy of its documentation and the study design and source of evidence should be carefully assessed. In terms of study design, randomized trials can be rated as providing high quality evidence and observational studies or case reports as low quality evidence. Further, several studies have cited the limitations of relying on product labels as the source of the evidence. Product labels are often over inclusive and may result in over inclusion of interactions that may not necessarily be clinically proven. Assessment of primary and secondary sources of literature, such as, the U.S. Food and Drug Administration (FDA) Adverse Event Reporting System (AERS) database of spontaneous adverse event reports should be included when assessing the evidence associated with an interaction. Seidling, et al. compared the dosage recommendations provided in product labels and found that dosage recommendations for specific DDIs are seldom provided in the drug label. When these authors evaluated the effect of the upper dose limits recommended in the drug labels on the frequency of alerts, they found that following recommendations from the published literature rather than the product labels could reduce the alert frequency by 55% [32]. Rigorous study designs offer better quality in making conclusions regarding an interaction. One study assessing the interaction between warfarin and acetaminophen described how observational studies were insufficient to make conclusions given the nonprescription or “over-the-counter” status of acetaminophen [38]. Thus, randomized studies allowed for the assessment of the temporal relationship, measurable effect with de-challenge and re-challenge, dose response, exclusion or accounting of other possible etiologic factors, and biological plausibility. Panelists pointed out the limitations in the existence of randomized trials for every member in a class of drugs thus resulting in extrapolation across a class rather than specific assessment to ascertain the biological plausibility of the interaction for each member within the class. Gold standard studies do not exist and quantity of evidence is sometimes limited to case reports and not controlled studies. KB vendors identified that there is limited availability of primary literature to assess the likelihood of an interaction compelling them to rely on package inserts which are inaccurate, have large inconsistencies across countries, out-dated and do not always match with the medical literature. The panel suggested that information from FDA, international regulatory agencies and treatment guidelines are a good resource for confirming if drugs are safe to co-prescribe. Providing this information in an abbreviated manner within the alert rather than expecting the provider to read the resources at the time of clinical decision making, would also improve assessment of the evidence in order to make appropriate therapeutic decisions regarding a DDI.

## Discussion

Current medication knowledge bases have overly sensitive rules causing excessive alerts and several design and implementation methods can be taken to reduce the number of disruptive alerts. Using a two pronged approach using a literature review and then validating the findings with an expert panel brought deep insight on various criteria that need to be considered identifying critical DDIs for use in EHRs.

Consideration of patient characteristics in combination with drug characteristics (called probability of an interaction) by far represented the most important criteria. The interplay between these two criteria provided a good risk assessment of the interaction. Panelists suggested several physician related strategies for reducing alert fatigue, such as assessing physician’s previous responses to the alert, consideration of the workflow and customizing alerts based on physician practice, knowledge and comfort level [39]. Another suggestion was the suppression of DDI alerts for drugs within order sets and treatment guidelines since the appropriateness for these would have been agreed upon previously by physicians. However the panel agreed that limiting certain levels of alerts to physicians was not a good idea and not showing the same alerts to pharmacists and physicians may create challenges in communication. They felt that management options, including monitoring approaches, do not change the significance of a potential interaction, for, in spite of overriding an alert the clinician might actually take relevant clinical or biochemical monitoring measures. Thus, they believed that this information should be included in the alert message but should not be used to determine the severity of the interaction.

The panel felt that periodic reviews are important as they would result in addition of new alerts and adjustment of previous alerts, based on alert action data, as new evidence regarding drugs becomes available or new drugs enter the market. They also acknowledge that such quality improvement efforts for the maintenance of a knowledge base required a considerable undertaking and was a highly resource intensive process. Panelists discussed the KB developed in the Netherlands by The Royal Dutch Association for the Advancement of Pharmacy as an example of a nationally implemented KB and advised others to leverage their work in order to decrease the labor needed to create such a list [40].

Patient characteristics allow the provider to determine the relevance of the risk of the DDI in the specific patient context. While this information regarding the patient characteristics relevant to a DDI is readily available, several barriers exist in not being able to readily adopt it in the context of improving alert specificity. Some vendors described their frustration at the lack of support from EMR vendors or hospital sites in the implementation of the patient contextualized logic used for DDI alerting. Since integration of patient characteristics in the CDS system is a resource intensive process and we do not have adequate technological support to incorporate these additional features this parameter should be considered for development of more sophisticated decision support systems in the future.

Another barrier is the lack of standardization of definitions due to localized definitions and customized ranges used by providers. For example, if a certain interaction was relevant only in the context of patients with renal failure then KB vendors need a standardized definition of what is meant by “renal failure” so that this logic can be programmatically implemented. Clinicians vary in their interpretations of this logic and often localization of such clinical definitions inhibits the incorporation of such logic into decision support rules. Further, even if there were standardization of such logic, its incorporation in the knowledge base would require strong linkage between the EHR and the KB to combine the relevant data and logic to make a refined, patient-specific recommendation, though such linkages have many benefits [41]. In an environment where the above barriers exist, participants suggested that key pieces of information should be included in the textual information contained in an alert at least initially.

The second criterion which was considered most important was an understanding of the drug characteristics, which we called the “probability of the interaction” in our discussion. This criterion takes into account considerations of the duration of therapy, dosage, route, regimen, etc. Similar to patient characteristics, information about the influence of specific drug or regimen characteristics is available in medication knowledge bases but has not been readily implemented in the decision support systems. Participants discussed ways this information could be employed more effectively, such as, the timing, formulation and sequence of administration could be considered within the realm of the medication administration record. For example, an interaction between itraconazole with proton pump inhibitors occurs in capsule form but not the solution. Thus, in this example, in the context of an order entry system, a provider who chooses the solution should not receive the alert whereas the provider who chooses the capsule should.

The consideration of the context of the setting is important as availability of facilities for monitoring such as anticoagulation clinics, etc. could further lower the probability that a patient would actually have the interaction. Thus, allowing healthcare institutions or providers to manipulate the level of the interaction based on the consideration of the setting could provide another mechanism for assessing the priority of an interaction, though this may not be practical in the near term.

Lastly, development of a standardized scale with transparent criteria or editorial guidelines for evaluation of the evidence related to an interaction is needed. One example was the work that has been undertaken in the Netherlands where articles are categorized on a scale that allows scoring the evidence using explicit criteria. The scale also allows for coding extrapolated evidence in a manner that is distinguishable from evidence that has been gathered from empirical data. This allows transparency into the scoring of the evidence and uniformity for comparison for the adjudication of the level of an alert. When there is a lack of reliable clinical evidence related to an interaction, participants suggested consideration of the likelihood of an interaction based on the pharmacodynamic and pharmacokinetic properties. The value of quantity of evidence was undermined in light of such a consideration. Added to this discussion were the consideration of a pharmacologic mechanism class rather than the simplistic concept of a drug class. This would allow consideration of the pharmacokinetic properties to determine possible interactions even in the absence of published evidence (i.e. biological plausibility).

## Conclusions

This study describes the complexity of assessing DDIs for clinical decision support in EHRs. This is a first attempt to provide a comprehensive assessment of the literature and validate the criteria with an expert panel of knowledge base vendors. This approach enabled the insights discussed in the study to be pragmatic solutions for overcoming existing barriers to improve the specificity of DDI alerts in EHRs. While this represents a first step in identifying criteria and barriers for evaluating critical DDIs, future research is needed to develop standardized editorial guidelines for uniform adoption of these criteria across medication knowledge bases in EHRs.

## Abbreviations

ADE: Adverse Drug Event; CDS: Clinical Decision Support; DDI: Drug-Dug Interaction; EHR: Electronic Health Record; FDA: Food and Drug Administration; KB: Knowledge Base; MDS: Medication Decision Support; ONC-HIT: Office of the National Coordinator-Health Information Technology; SME: Subject Matter Expert.

## Competing interests

The authors declare that they have no competing interests.

## Authors' contributions

SP, AD, DB, DWB, and BM conceived the study idea and design. AD, SP, JD, and EY were responsible for data collection for the systematic review and the interpretation of data. SP, AD, MC, AC, and AT participated in writing and DB, DWB, and BM participated in revising the manuscript. All authors read and approved the final manuscript.

## Pre-publication history

The pre-publication history for this paper can be accessed here:

http://www.biomedcentral.com/1472-6947/13/65/prepub

## Supplementary Material

Additional file 1 Contains the final list of articles reviewed, and used to annotate tables 1 and 2, to determine both criteria and barriers needed for assessing high-priority DDIs. Click here for file
