# Supplementary material for: Criteria for assessing high-priority drug-drug interactions for clinical decision support in electronic health records
Source: BMC Med Inform Decis Mak. 2013 Jun 13;13:65. doi: 10.1186/1472-6947-13-65 (PMC3706355; doi:10.1186/1472-6947-13-65)
Supplement: Additional file 1 — Contains the final list of articles reviewed, and used to annotate tables 1 and 2, to determine both criteria and barriers needed for assessing high-priority DDIs. [file 1472-6947-13-65-S1.docx]

Additional file 1

[[[1-43](#_ENREF_1)]]

References

1. Abookire SA, Teich JM, Sandige H, Paterno MD, Martin MT, Kuperman GJ, Bates DW: **Improving allergy alerting in a computerized physician order entry system**. *Proceedings / AMIA Annual Symposium AMIA Symposium* 2000:2-6.

2. Agostini JV, Concato J, Inouye SK: **Improving sedative-hypnotic prescribing in older hospitalized patients: provider-perceived benefits and barriers of a computer-based reminder**. *Journal of general internal medicine* 2008, **23 Suppl 1**:32-36.

3. Ahearn MD, Kerr SJ: **General practitioners' perceptions of the pharmaceutical decision-support tools in their prescribing software**. *The Medical journal of Australia* 2003, **179**(1):34-37.

4. Ashworth M: **Re: GPs' views on computerized drug interaction alerts**. *Journal of clinical pharmacy and therapeutics* 2002, **27**(5):311-312.

5. Bates DW, Kuperman GJ, Wang S, Gandhi T, Kittler A, Volk L, Spurr C, Khorasani R, Tanasijevic M, Middleton B: **Ten commandments for effective clinical decision support: making the practice of evidence-based medicine a reality**. *Journal of the American Medical Informatics Association : JAMIA* 2003, **10**(6):523-530.

6. Bates DW, O'Neil AC, Boyle D, Teich J, Chertow GM, Komaroff AL, Brennan TA: **Potential identifiability and preventability of adverse events using information systems**. *Journal of the American Medical Informatics Association : JAMIA* 1994, **1**(5):404-411.

7. Berner ES, Kasiraman RK, Yu F, Ray MN, Houston TK: **Data quality in the outpatient setting: impact on clinical decision support systems**. *AMIA Annual Symposium proceedings / AMIA Symposium AMIA Symposium* 2005:41-45.

8. Bertsche T, Pfaff J, Schiller P, Kaltschmidt J, Pruszydlo MG, Stremmel W, Walter-Sack I, Haefeli WE, Encke J: **Prevention of adverse drug reactions in intensive care patients by personal intervention based on an electronic clinical decision support system**. *Intensive care medicine* 2010, **36**(4):665-672.

9. Chaffee BW, Zimmerman CR: **Developing and implementing clinical decision support for use in a computerized prescriber-order-entry system**. *American journal of health-system pharmacy : AJHP : official journal of the American Society of Health-System Pharmacists* 2010, **67**(5):391-400.

10. Chazard E, Ficheur G, Merlin B, Serrot E, Beuscart R: **Adverse drug events prevention rules: multi-site evaluation of rules from various sources**. *Studies in health technology and informatics* 2009, **148**:102-111.

11. Del Fiol G, Rocha BH, Kuperman GJ, Bates DW, Nohama P: **Comparison of two knowledge bases on the detection of drug-drug interactions**. *Proceedings / AMIA Annual Symposium AMIA Symposium* 2000:171-175.

12. Feldstein A, Simon SR, Schneider J, Krall M, Laferriere D, Smith DH, Sittig DF, Soumerai SB: **How to design computerized alerts to safe prescribing practices**. *Joint Commission journal on quality and safety* 2004, **30**(11):602-613.

13. Galanter WL, Didomenico RJ, Polikaitis A: **A trial of automated decision support alerts for contraindicated medications using computerized physician order entry**. *Journal of the American Medical Informatics Association : JAMIA* 2005, **12**(3):269-274.

14. Galanter WL, Hier DB, Jao C, Sarne D: **Computerized physician order entry of medications and clinical decision support can improve problem list documentation compliance**. *International journal of medical informatics* 2010, **79**(5):332-338.

15. Galanter WL, Polikaitis A, DiDomenico RJ: **A trial of automated safety alerts for inpatient digoxin use with computerized physician order entry**. *Journal of the American Medical Informatics Association : JAMIA* 2004, **11**(4):270-277.

16. Glassman PA, Simon B, Belperio P, Lanto A: **Improving recognition of drug interactions: benefits and barriers to using automated drug alerts**. *Medical care* 2002, **40**(12):1161-1171.

17. Grizzle AJ, Mahmood MH, Ko Y, Murphy JE, Armstrong EP, Skrepnek GH, Jones WN, Schepers GP, Nichol WP, Houranieh A, Dare DC, Hoey CT, Malone DC: **Reasons provided by prescribers when overriding drug-drug interaction alerts**. *The American journal of managed care* 2007, **13**(10):573-578.

18. Hansten PD, Horn JR, Hazlet TK: **ORCA: OpeRational ClassificAtion of drug interactions**. *J Am Pharm Assoc (Wash)* 2001, **41**(2):161-165.

19. Isaac T, Weissman JS, Davis RB, Massagli M, Cyrulik A, Sands DZ, Weingart SN: **Overrides of medication alerts in ambulatory care**. *Archives of internal medicine* 2009, **169**(3):305-311.

20. Judge J, Field TS, DeFlorio M, Laprino J, Auger J, Rochon P, Bates DW, Gurwitz JH: **Prescribers' responses to alerts during medication ordering in the long term care setting**. *Journal of the American Medical Informatics Association : JAMIA* 2006, **13**(4):385-390.

21. Kawamoto K, Houlihan CA, Balas EA, Lobach DF: **Improving clinical practice using clinical decision support systems: a systematic review of trials to identify features critical to success**. *BMJ* 2005, **330**(7494):765.

22. Ko Y, Abarca J, Malone DC, Dare DC, Geraets D, Houranieh A, Jones WN, Nichol WP, Schepers GP, Wilhardt M: **Practitioners' views on computerized drug-drug interaction alerts in the VA system**. *Journal of the American Medical Informatics Association : JAMIA* 2007, **14**(1):56-64.

23. Krall MA, Sittig DF: **Clinician's assessments of outpatient electronic medical record alert and reminder usability and usefulness requirements**. *Proceedings / AMIA Annual Symposium AMIA Symposium* 2002:400-404.

24. Magnus D, Rodgers S, Avery AJ: **GPs' views on computerized drug interaction alerts: questionnaire survey**. *Journal of clinical pharmacy and therapeutics* 2002, **27**(5):377-382.

25. Malone DC, Abarca J, Hansten PD, Grizzle AJ, Armstrong EP, Van Bergen RC, Duncan-Edgar BS, Solomon SL, Lipton RB: **Identification of serious drug-drug interactions: results of the partnership to prevent drug-drug interactions**. *Journal of the American Pharmacists Association : JAPhA* 2004, **44**(2):142-151.

26. Malone DC, Hutchins DS, Haupert H, Hansten P, Duncan B, Van Bergen RC, Solomon SL, Lipton RB: **Assessment of potential drug-drug interactions with a prescription claims database**. *American journal of health-system pharmacy : AJHP : official journal of the American Society of Health-System Pharmacists* 2005, **62**(19):1983-1991.

27. Mille F, Schwartz C, Brion F, Fontan JE, Bourdon O, Degoulet P, Jaulent MC: **Analysis of overridden alerts in a drug-drug interaction detection system**. *International journal for quality in health care : journal of the International Society for Quality in Health Care / ISQua* 2008, **20**(6):400-405.

28. Murphy JE, Malone DC, Olson BM, Grizzle AJ, Armstrong EP, Skrepnek GH: **Development of computerized alerts with management strategies for 25 serious drug-drug interactions**. *American journal of health-system pharmacy : AJHP : official journal of the American Society of Health-System Pharmacists* 2009, **66**(1):38-44.

29. Nightingale PG, Adu D, Richards NT, Peters M: **Implementation of rules based computerised bedside prescribing and administration: intervention study**. *BMJ* 2000, **320**(7237):750-753.

30. Paterno MD, Maviglia SM, Gorman PN, Seger DL, Yoshida E, Seger AC, Bates DW, Gandhi TK: **Tiering drug-drug interaction alerts by severity increases compliance rates**. *Journal of the American Medical Informatics Association : JAMIA* 2009, **16**(1):40-46.

31. Seidling HM, Schmitt SP, Bruckner T, Kaltschmidt J, Pruszydlo MG, Senger C, Bertsche T, Walter-Sack I, Haefeli WE: **Patient-specific electronic decision support reduces prescription of excessive doses**. *Quality & safety in health care* 2010, **19**(5):e15.

32. Shah NR, Seger AC, Seger DL, Fiskio JM, Kuperman GJ, Blumenfeld B, Recklet EG, Bates DW, Gandhi TK: **Improving acceptance of computerized prescribing alerts in ambulatory care**. *Journal of the American Medical Informatics Association : JAMIA* 2006, **13**(1):5-11.

33. Strom BL, Schinnar R, Bilker W, Hennessy S, Leonard CE, Pifer E: **Randomized clinical trial of a customized electronic alert requiring an affirmative response compared to a control group receiving a commercial passive CPOE alert: NSAID--warfarin co-prescribing as a test case**. *Journal of the American Medical Informatics Association : JAMIA* 2010, **17**(4):411-415.

34. Tamblyn R, Huang A, Taylor L, Kawasumi Y, Bartlett G, Grad R, Jacques A, Dawes M, Abrahamowicz M, Perreault R, Winslade N, Poissant L, Pinsonneault A: **A randomized trial of the effectiveness of on-demand versus computer-triggered drug decision support in primary care**. *Journal of the American Medical Informatics Association : JAMIA* 2008, **15**(4):430-438.

35. Taylor LK, Tamblyn R: **Reasons for physician non-adherence to electronic drug alerts**. *Studies in health technology and informatics* 2004, **107**(Pt 2):1101-1105.

36. van der Sijs H, Aarts J, van Gelder T, Berg M, Vulto A: **Turning off frequently overridden drug alerts: limited opportunities for doing it safely**. *Journal of the American Medical Informatics Association : JAMIA* 2008, **15**(4):439-448.

37. van der Sijs H, Aarts J, Vulto A, Berg M: **Overriding of drug safety alerts in computerized physician order entry**. *Journal of the American Medical Informatics Association : JAMIA* 2006, **13**(2):138-147.

38. van der Sijs H, Bouamar R, van Gelder T, Aarts J, Berg M, Vulto A: **Functionality test for drug safety alerting in computerized physician order entry systems**. *International journal of medical informatics* 2010, **79**(4):243-251.

39. van der Sijs H, Kowlesar R, Klootwijk AP, Nelwan SP, Vulto AG, van Gelder T: **Clinically relevant QTc prolongation due to overridden drug-drug interaction alerts: a retrospective cohort study**. *British journal of clinical pharmacology* 2009, **67**(3):347-354.

40. van Roon EN, Flikweert S, le Comte M, Langendijk PN, Kwee-Zuiderwijk WJ, Smits P, Brouwers JR: **Clinical relevance of drug-drug interactions : a structured assessment procedure**. *Drug safety : an international journal of medical toxicology and drug experience* 2005, **28**(12):1131-1139.

41. Vaziri A, Connor E, Shepherd I, Jones RT, Chan T, de Lusignan S: **Are we setting about improving the safety of computerised prescribing in the right way? A workshop report**. *Informatics in primary care* 2009, **17**(3):175-182.

42. Weingart SN, Toth M, Sands DZ, Aronson MD, Davis RB, Phillips RS: **Physicians' decisions to override computerized drug alerts in primary care**. *Archives of internal medicine* 2003, **163**(21):2625-2631.

43. Payne TH, Nichol WP, Hoey P, Savarino J: **Characteristics and override rates of order checks in a practitioner order entry system**. *Proceedings / AMIA Annual Symposium AMIA Symposium* 2002:602-606.

_________
